# The SL-I structural element of the 3′ UTR region of West Nile virus participates in the regulation of viral translation

**DOI:** 10.1099/jgv.0.002268

**Published:** 2026-05-28

**Authors:** Cristina Romero-López, Pilar Bueno-Arribas, Alfredo Berzal-Herranz

**Affiliations:** 1 Instituto de Parasitología y Biomedicina “López-Neyra”, IPBLN-CSIC, PTS Granada, Av. del Conocimiento 17, 18016, Armilla (Granada), Spain

**Keywords:** 3′ UTR, RNA genome, West Nile virus (WNV), West Nile virus translation regulation

## Abstract

The genome of West Nile virus (WNV) is a positive, ssRNA molecule, which encodes a single ORF flanked by UTRs (5′ and 3′ UTRs). These UTRs are enriched with structural RNA elements that play critical roles in the viral cycle, including translation, replication and encapsidation. The 3′ UTR is crucial for translation control by the combined enhancer and repressor activities of different structural elements. This manuscript provides new roles for the 3′ UTR SL-I element as a translation inhibitor in WNV in cis. This inhibitory effect is primarily observed in a cap-dependent translation context, most likely affecting multi-round translation efficiency. The molecular mechanism underlying this phenomenon appears to involve the recruitment of the 40S ribosomal subunit by the 3′ UTR. We have previously demonstrated that the 3′ UTR recruits the 40S subunit at two independent sites and influences its proper positioning at the 5′ UTR. The present work identifies the SL-I element as a critical regulator of WNV translation at late stages of protein synthesis. This can be due, at least partially, to the modulation of the 40S positioning at the 3′ UTR, as detected by selective 2′-hydroxyl acylation analysed by primer extension (SHAPE) analyses. These findings provide new insights into the role of the 3′ UTR SL-I element in translation regulation and suggest that WNV translation is a finely tuned process dependent on the coordinated and well-balanced interplay of multiple partners.

## Graphical Abstract

The SL-I element of the 3′ UTR of WNV regulates the 40S recruitment, modulating the affinity kinetics of its interaction and its positioning at the genomic 3′ UTR.

## Introduction

West Nile virus (WNV) is a member of the genus *Orthoflavivirus*, family *Flaviviridae*. This genus includes many important infectious agents besides WNV, such as dengue virus (DENV), Zika virus (ZIKV) or yellow fever virus (YFV), which are transmitted by hematophagous arthropod vectors. WNV is mainly transmitted to birds through the bite of infected mosquitoes, mainly of the genus *Culex* sp., while humans and horses are considered accidental hosts that act as a dead-end, thus preventing further transmission [1].

The genome of orthoflaviviruses is a positive-sense, ssRNA molecule that contains a type I cap at the 5′ end and lacks a poly(A) tail [2]. Instead, orthoflaviviruses possess a conserved CU dinucleotide at the 3′ end (CU-OH). The RNA genome encodes a single ORF flanked by UTRs at both the 5′ and 3′ termini. The 5′ UTR is relatively short (~100 nts) and highly conserved among the isolates of a specific viral species, in both sequence and secondary structure, whereas the 3′ UTR exhibits greater sequence variability (350–700 nts), particularly at its 5′ proximal tract. Both UTRs contain conserved structural RNA elements that regulate key steps of the viral life cycle, including translation and replication, as well as immune evasion and pathogenesis [3].

**Fig. 1.**
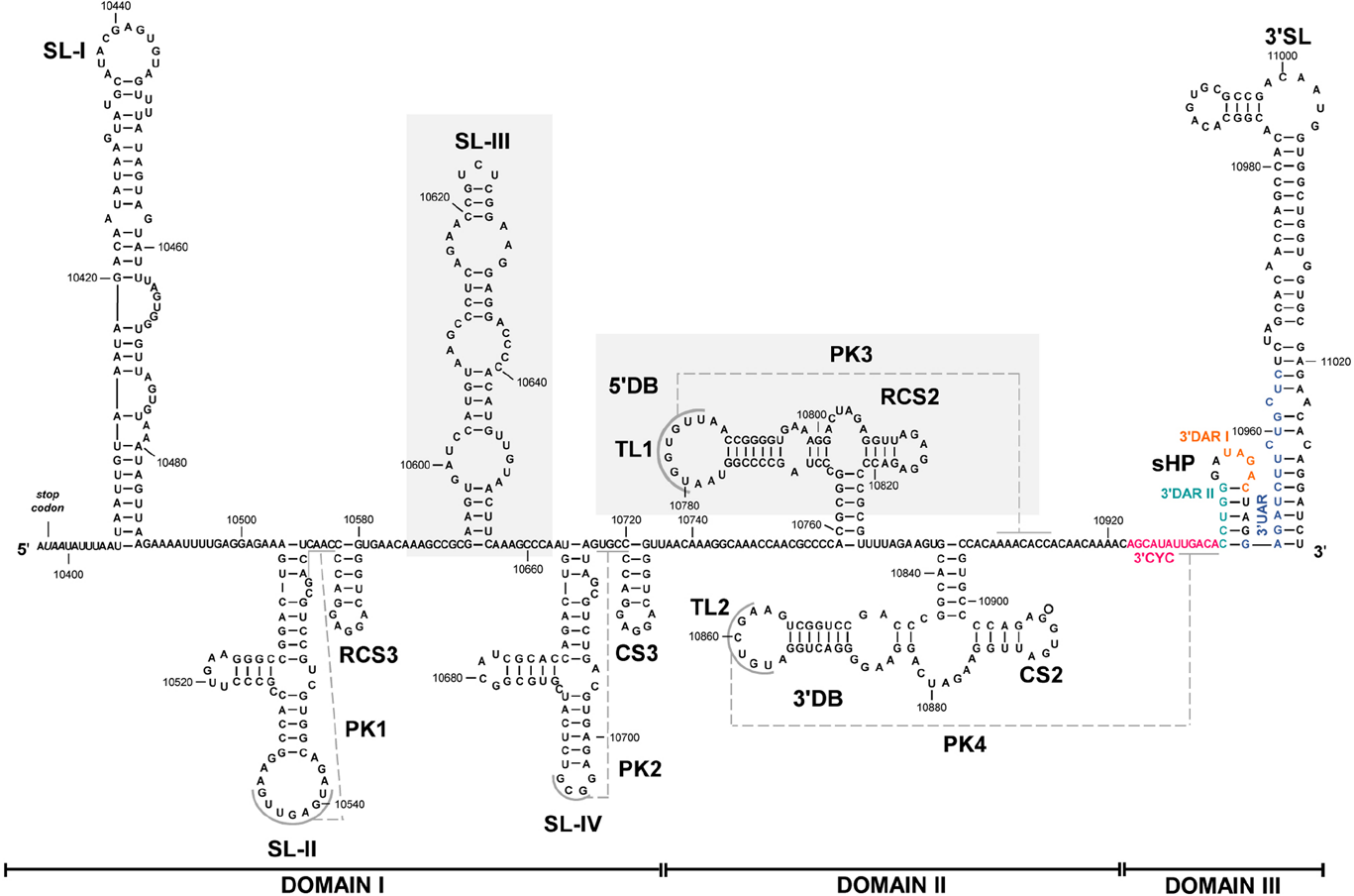
Sequence and secondary structure model proposed for the WNV 3′ UTR. The figure shows the sequence and the secondary structure proposed for the WNV 3′ UTR. The translation stop codon is shown in italics. Pseudoknots are indicated as PK1-PK4 and marked by dashed grey lines. Cyclization sequences 3′CYC, 3′DARI, 3′DAR II and 3′UAR are depicted in pink, orange, green and blue, respectively. Shadowed boxes indicate the recruiting sites for the 40S ribosomal subunit. CS, conserved sequence; RCS, repeated conserved sequence. Numbers refer to the nucleotide positions of the WNV NY99-flamingo382–99 strain [28] with the modifications reported (GeneBank access AF196835) [29].

The WNV 3′ UTR has traditionally been defined as composed of three structural domains (I–III; Fig. 1) [2]. (i) Domain I, located immediately downstream of the translation stop codon, comprises the SL-I–SL-IV elements, including the PK1 and PK2 pseudoknots. This domain is characterized by the presence of the duplicated cassettes SL-I–SL-II–PK1–RCS3 and SL-III–SL-IV–PK2–CS3. These duplications contribute to functional diversity by permitting sequence variability [4]. Indeed, domain I is the most sequence-variable region among different WNV isolates, with the SL-I element showing the lowest level of conservation. SL-I is defined as a UAG-rich region that has been associated with the binding of the Musashi protein [5], a known regulator of translation control. Nevertheless, the precise role of SL-I in the viral cycle remains unclear. (ii) Domain II contains the duplicated 5′ and 3′ dumbbell (DB) elements, involved in the formation of the PK3 and PK4 pseudoknots. Both pseudoknots are critical for viral replication and act to enhance translation [6,9]. (iii) Domain III is the most structurally conserved domain of the 3′ UTR. It includes the small hairpin (sHP) and the 3′ stem-loop (3′SL) elements, both of which are required for viral replication [10,13] and translation [8,14, 15]. Notably, the structural boundaries of domains I–III have recently been redefined. Emerging evidence suggests that the 3′ end of the ORF may contribute to the 3′ UTR organization, enabling long-range RNA–RNA interactions that promote a dynamic three-dimensional conformation of the 3′ UTR [16].

The folding, and hence the function, of the 3′ UTR is modulated by the genomic RNA cyclization. This process involves the establishment of long-range RNA–RNA interactions between complementary sequences located at the 5′ and 3′ ends of the viral genome, specifically the UAR, CYC and DAR motifs. These interactions drive the formation of a circular conformation of the genomic RNA. Cyclization is essential for the initiation of viral RNA replication [17,21]. However, this conformation can interfere with translation initiation by disrupting the proper folding of structural RNA elements required for efficient ribosomal scanning, such as the cHP [22]. We have recently provided evidence indicating that the joint action of both genomic ends contributes to the positioning of the 40S subunit at the translation initiation site [8].

It is widely accepted that the 3′ UTR enhances translation efficiency in orthoflaviviruses [6,7, 23]. During the early stages of infection, the WNV genome functions as an mRNA that is actively translated to produce both structural and non-structural viral proteins. Translation initiation occurs primarily through a cap-dependent mechanism. Cap-independent translation initiation has also been described in DENV, WNV, ZIKV and other orthoflaviviruses, particularly under conditions of cellular stress that inhibit global protein synthesis [6,24,26]. Although the precise molecular mechanisms governing the switch between cap-dependent and cap-independent translation remain unclear, it has been demonstrated that conserved elements within the 3′ UTR are required for both processes [6].

Translation control is mediated by the coordinated activity of various elements within the WNV 3′ UTR [6,8]. These elements can exert opposing effects – either enhancing or inhibiting translation – thereby compensating for one another and conferring multifunctional regulatory capacity to the 3′ UTR. One of the mechanisms underlying this regulation involves the differential recruitment of the 40S ribosomal subunit by the 5′DB and SL-III elements [8]. The efficient 3′ UTR-40S interaction also requires the formation of the PK3–PK4 pseudoknots. The 40S recruitment via the 5′DB element is characterized by high-affinity binding and has been associated with the correct positioning of the 40S subunit at the 5′ UTR, whereas binding through SL-III occurs with lower affinity and may be involved in sequestering components of the translation machinery and controlling ribosomal subunit recycling. This dual functionality of the 3′ UTR enables regulation of translation at both initiation and termination stages, thereby contributing to global regulation of the process. Thus, the 40S ribosomal subunit would act as a bridge to facilitate cross-talk between both ends of the viral genome, similarly to the circularization of eukaryotic mRNAs during translation [27].

The present study provides evidence that SL-I functions as a key regulator of translational control. Similar to SL-III, SL-I acts by reducing overall translation efficiency, likely by interfering with the recruitment of the 40S ribosomal subunit via the 5′DB element and by promoting conformational changes in the PK3 and PK4 pseudoknots. These findings support a model in which translational regulation in WNV is mediated by the coordinated action of multiple 3′ UTR elements that exert opposing effects. Such a mechanism may serve to buffer fluctuations within the system and facilitate transitions between distinct stages of the viral life cycle.

## Methods

### DNA transcription templates and RNA constructs

The WNV genomic RNA fragments used in this study derive from the WNV NY99-flamingo382–99 strain [28] with the modifications reported (GeneBank access AF196835) [29].

DNA template encoding the WNV RNA construct was obtained as described [6] from the plasmid pGLWNV linearized with *Bam*HI. This plasmid codes the 5′ end of the WNV genome, including the 5′ UTR plus the cHP element (156 nts) and the entire 3′ UTR flanking the FLuc coding sequence, which is in phase with the WNV AUG located in the 5′ UTR. Linearization of pGLWNV with *Xba*I yielded the template for the synthesis of the RNA construct WNVΔ3′UTR [6].

The plasmid encoding the WNV_dSLI construct, pGLWNV_dSLI, was obtained by site-directed mutagenesis from the plasmid pGLWNV, using the Phusion Site-Directed Mutagenesis Kit (Thermo Fisher Scientific, Waltham, MA, USA) and the appropriate primers, as described in [30] (see Table S1, available in the online Supplementary Material). This plasmid resembles the parental construct but lacks the 3′ UTR SL-I element coding sequence (nts 10,400 to 10,500). pGLWNV_dSLI was digested with *Bam*HI to generate the DNA template for the synthesis of the RNA WNV_dSLI. The DNA template encoding the construct WNV_dSLIII was obtained by *Bam*HI digestion of the plasmid pGLWNV_dSLIII, as described [6].

The plasmid pGLDENV, encoding the DENV construct, was obtained by recombination using the In-Fusion® HD Cloning Plus kit from Takara (Kusatsu, Japan), following the manufacturer’s instructions. This plasmid contains the FLuc coding sequence flanked by the first 161 nts of the DENV genome and the DENV 3′ UTR. The AUG start codon of FLuc was deleted to ensure translation initiation from the viral AUG codon. The fragment encoding the 5′ DENV sequence (1–161 nts) from lineage 2 (GeneBank access NC_001474.2) was purchased from Integrated DNA Technologies (Coralville, IA, USA). Briefly, the plasmid pGLWNV-3′ DENV [6] was digested with *Kpn*I and *Hind*III to remove the region encoding the WNV 5′ end. The 5′ DENV fragment was then introduced by recombination between the *Kpn*I and *Hind*III restriction sites to generate pGLDENV.

Plasmids encoding DENV+SLI or DENV+SLIII constructs were derived from pGLDENV by replacing the 3′ UTR with the chimeric constructs comprising the DENV 3′ UTR plus the WNV SL-I or SL-III structural elements, positioned according to their native relative locations in the WNV 3′ UTR. These plasmids were generated by recombination as described above. The pGLDENV plasmid was digested with *Bam*HI and *Sal*I to replace the DENV 3′ UTR with the chimeric fragments, generating the pGLDENV+SLI or pGLDENV+SLIII plasmids. Digestion of pGLDENV, pGLDENV+SLI or pGLDENV+SLIII with *Sal*I yielded linearized templates for RNA synthesis. Digestion of pGLDENV with *Bam*HI was used to generate the DNA template for the synthesis of the DENVΔ3′UTR RNA.

The DNA encoding the m^7^G-RLuc RNA was obtained by linearization of the plasmid pRLSV40 (Promega, Madison, WI, USA) with *Bam*HI, as described [31]. The DNA templates encoding the constructs WNV 3′ UTR and RNA100 were generated as previously described [32,33].

All the RNA constructs were obtained by *in vitro* transcription using the HighYield T7 RNA Synthesis kit (Jena Bioscience, Jena, Germany). The 5′ cap (m^7^G) was incorporated during RNA synthesis as described [30]. Biotinylated RNAs, b-WNV-3′UTR and b-3′WNV_dSLI, were obtained by *in vitro* transcription from their corresponding DNA templates and were internally biotinylated (b) by using biotin-16-UTP, as previously described [34]. The amount of biotin-UTP was calculated to allow for the incorporation of one biotin residue per molecule. RNAs were purified and quantified as previously reported [30]. Briefly, following transcription, RNA molecules were purified by phenol extraction, followed by chloroform:isoamyl alcohol (24:1) extraction. Unincorporated nucleotides were removed by two consecutive steps of size exclusion chromatography (Sephadex G-25, GE Healthcare). RNA concentration was determined by A_260_ measurements in a NanoDrop™ spectrophotometer, and the extent of protein and carbohydrate/phenolic contaminations was assessed by A_260_/A_280_ and A_260_/A_230_ ratios, respectively. The integrity of the RNA was evaluated by denaturing agarose-formaldehyde gel electrophoresis.

Primer sequences used in this study are shown in Table S1.

### Cell culture

Vero cells derived from African green monkey kidney were maintained in Minimum Essential Medium (MEM) supplemented with 5% of heat-inactivated FBS (Gibco® by Life Technologies™, Invitrogen, Waltham, MA, USA), 2 mM l-glutamine (Sigma, St. Louis, MO, USA) and 1 mM sodium pyruvate (Sigma) at 37 °C in a 5% CO_2_ atmosphere.

### S10 cell lysate production and purification of 40S ribosomal subunits

S10 Vero cell lysates for *in vitro* translation assays were prepared as previously described [35] with slight modifications. Briefly, 2.5×10^8^ cells were grown as described above to reach 100% confluency. Then, cells were subjected to trypsin treatment, washed twice with ten volumes of isotonic buffer (35 mM HEPES/KOH, pH 7.6, 150 mM NaCl and 11 mM glucose) and pelleted by centrifugation at 100 ***g*** during 5 min at 4 °C. Lysis was initiated by gentle resuspension of the pellet in 1.5 vol of hypotonic buffer (20 mM HEPES/KOH, pH 7.6, 10 mM KCl, 1.5 mM MgOAc, 1 mM DTT) supplemented with cOmplete™ protease inhibitor (Merck KGaA, Darmstadt, Germany) at 4 °C. The mixture was incubated on ice for 20 min. Then, the cell lysate was homogenized in a Dounce homogenizer by the application of 25 strokes, equilibrated with 0.2 vol of S10 buffer (100 mM HEPES-KOH pH 7.6, 0.6 M KOAc, 20 mM MgOAc, 25 mM DTT and cOmplete™ protease inhibitor) and subjected to centrifugation at 10,000 ***g*** for 10 min at 4 °C, to eliminate cell debris. The A_280_ of the clear lysates was determined to assess an optimal protein concentration of ~30–40 µg µl^−1^.

For the purification of the 40S subunit, cell lysates were prepared as noted above with the modifications described in [8]. Briefly, S10 fractions obtained from 5×10^8^ Vero cells were layered onto 3 ml of a 1 M sucrose cushion prepared in buffer A (Tris-HCl, pH 7.6, 20 mM; DTT, 2 mM; MgCl_2_, 6 mM; and KCl, 0.5 M) and subjected to ultracentrifugation at 40,000 r.p.m. for 4 h at 4 °C in a Beckman 70.1 Ti rotor. The pellet containing polysomes was resuspended in buffer B (Tris-HCl, pH 7.6, 20 mM; DTT, 2 mM; MgCl_2_, 6 mM; and KCl, 150 mM) to a final concentration of 50–150 A_260_ units. This suspension was incubated with 4 mM puromycin for 10 min at 4 °C, followed by incubation at 37 °C for 30 min. Translation initiation factors bound to the ribosomal subunits were then released by the addition of 0.5 M KCl. The ribosomal subunits were loaded onto a continuous 10–45% linear sucrose gradient in buffer A and separated by ultracentrifugation in a Beckman SW40 rotor device at 28,000 r.p.m. overnight. Fractions of 500 µl were collected from the top of the gradient and concentrated using Amicon® Ultra-2 30 kDa. The concentration of the 40S subunits was calculated by UV spectrometry measurements according to 1 A_260_=30 pmol µl^−1^.

### Translation assays in cell culture

Vero cell transfection was performed essentially as described [6]. Briefly, 60,000 cells were seeded on a 24-well plate 24 h before transfection to achieve 80% confluency. RNA molecules were denatured by heating at 95 °C for 2 min and further cooling on ice for 15 min. Then, 1.5 µg of the construct under study and 300 ng of the m^7^G-RLuc RNA were mixed with 100 µl of Opti-MEM® (Gibco® elative by Life Technologies™) and 2 µl of TransFectin™ (Bio-Rad, Hercules, CA, USA) as transfection reagent and incubated at 25 °C for 10 min. The mixture was added to the cell culture and incubated for 6 h (or the indicated times for the kinetic analysis) at 37 °C. Then, cells were lysed and luciferase units were measured using the Dual-Luciferase Reporter Assay Kit (Vazyme, Nanjing, China), following the manufacturer’s instructions. Relative translation efficiency was calculated from the FLuc/RLuc ratio.

For the analysis of the SL-I effect on viral translation in trans, a mixture containing 1.5 µg of either m^7^G-WNV or m^7^G-WNV_dSLI, 300 ng of the m^7^G-RLuc, 100 µl of Opti-MEM® (Invitrogen) and 3 µl of TransFectin™ (Bio-Rad) was supplemented with 12.5 pmol of either the SL-I RNA or a non-related RNA, RNA100. The total amount of RNA was kept constant across all the transfections (1.5 µg), with a molar ratio 1 : 10 (m^7^G-WNV or m^7^G-WNV_dSLI:SL-I or RNA100). RNA molecules were denatured and renatured as described above prior to transfection. Cells were lysed and luciferase activity was measured as noted above.

### *In vitro* translation assays

*In vitro* translation assays were performed using Vero cell S10 extracts, essentially as described [36]. Briefly, 50 µl reactions were prepared with 25 µl of cell extract to reach a protein concentration ~15 µg µl^−1^. The lysates were supplemented with 20 mM HEPES-KOH, pH 7.5, 100 mM KOAc, 1.5 mM MgOAc, 1 mM ATP, 5 mM GTP, 9 mM creatine phosphate, 20 ng µl^−1^ creatine phosphokinase (Merck, Germany), 2 mM DTT, 200 nM spermidine, 10 nM amino acid mixture (Promega), 2 mM d-luciferin sodium salt (Merck) and 10 ng µl^−1^ of the RNA of interest. Reactions proceeded at 30 °C for 30 min in a 96-well white, flat-bottom plate. Relative luciferase units were determined at 20-s intervals throughout the reaction time to ensure continuous measurement of the emitted light quantity, using an Infinite F200 microplate reader (Tecan, Switzerland). The collected data were used to calculate the first-round translation constant as reported [37]. The global translation rate and the steady-state luciferase production were calculated by applying a Gompertz equation fitting *y* = *y*_0_ + a*e^(-(*x*_0_-*x*)/*b*)^ with SigmaPlot 15.0 software. At least three replicates of each assay were performed.

### RNA stability assays

Vero cells were cotransfected with the indicated RNA constructs as described above. At 6 h post-transfection, cells were washed with PBS 1X and total RNA was isolated using TRIzol reagent (Thermo Fisher Scientific), as previously described [38]. RNA quality was monitored by gel electrophoresis in a 1% agarose gel and quantified by densitometry. Relative amount of the RNA constructs under study were determined by reverse transcription-quantitative PCR (RT-qPCR) and normalized to those obtained for the GAPDH mRNA, as reported [38]. Briefly, 200 ng of RNA was hybridized with 2 µg of random primers by heating at 95 °C and cooling up to 16 °C. MultiScribe reverse transcriptase was then added to a final concentration of 0.5 U µl^−1^ and primer extension proceeded for 30 min at 16 °C and an additional extension step at 37 °C for 30 min. Reaction was stopped by heating at 85 °C for 5 min. A fraction corresponding to one-sixth of the reaction mix was used for the next qPCR step using TB Green® Premix Ex Taq™ II FAST qPCR (Takara Bio, San José, CA, USA) and amplified over 40 cycles using specific primers. PCR was performed in a CFX96 real-time PCR detection system (Bio-Rad). The results were analysed using Bio-Rad CFX Manager (Bio-Rad).

### Structural selective 2′-hydroxyl acylation analysed by primer extension analysis

Selective 2′-hydroxyl acylation analysed by primer extension (SHAPE) analyses of the m^7^G-WNV and m^7^G-WNV_dSLI RNAs were performed both in the absence and presence of the 40S ribosomal subunit. Briefly, 5 pmol of the RNA under study was denatured by heating at 95 °C for 2 min, followed by snap cooling on ice for 15 min. Renaturation was carried out by incubating the RNA at 37 °C for 10 min in SHAPE buffer (20 mM HEPES-NaOH, pH 7.5, 100 mM NaCl and 1 mM MgCl₂). When indicated, a tenfold molar excess of the 40S ribosomal subunit was added prior to chemical probing, which was initiated by the addition of 15 mM *N*-methylisatoic anhydride (NMIA) in DMSO. Reactions were incubated for 30 min at 37 °C. Control reactions containing only the probe solvent (DMSO) were performed in parallel. Chemical modification was quenched by the addition of cold 0.3 M NaOAc, pH 5.2. RNAs were then ethanol-precipitated and subjected to primer extension to identify modified nucleotides, as previously described [8]. Reverse transcription reactions were stopped by treatment with 0.2 M NaOH for 5 min at 95 °C, followed by neutralization with unbuffered 0.5 M Tris-HCl, as described elsewhere [36]. cDNAs were resolved by capillary electrophoresis, and electropherograms were analysed using the QuShape software [37].

The obtained relative SHAPE reactivities were processed to enable comparison across experimental conditions, following established methods [39]. A reference nucleotide track (A_10752_-C_10756_) showing positive reactivity (>0.35 SHAPE units) and minimal variation among constructs was selected. The average reactivity of these nucleotides was used to normalize individual nucleotide reactivities across datasets. Normalized values were subsequently scaled, and a threshold reactivity value was defined as Q3+IQR, corresponding to 0.32 units. While this approach does not provide information at the level of electronic dynamics, it offers a robust framework for comparative analysis of RNA structural flexibility across different molecular contexts.

### Statistical methods

Data are presented as means±sd of at least three independent experiments, unless otherwise indicated. Pairwise comparisons were conducted using the unpaired two-tailed Mann–Whitney test [40]. In addition, the Kruskal–Wallis test was applied to assess statistically significant differences among three or more conditions in the SHAPE analyses. Significance was set at *P*≤0.05. When required, *P* value was fitted by the Benjamini–Hochberg method to reduce the emergence of false-positive data.

## Results

### SL-I negatively regulates WNV cap-dependent translation

**Fig. 2.**
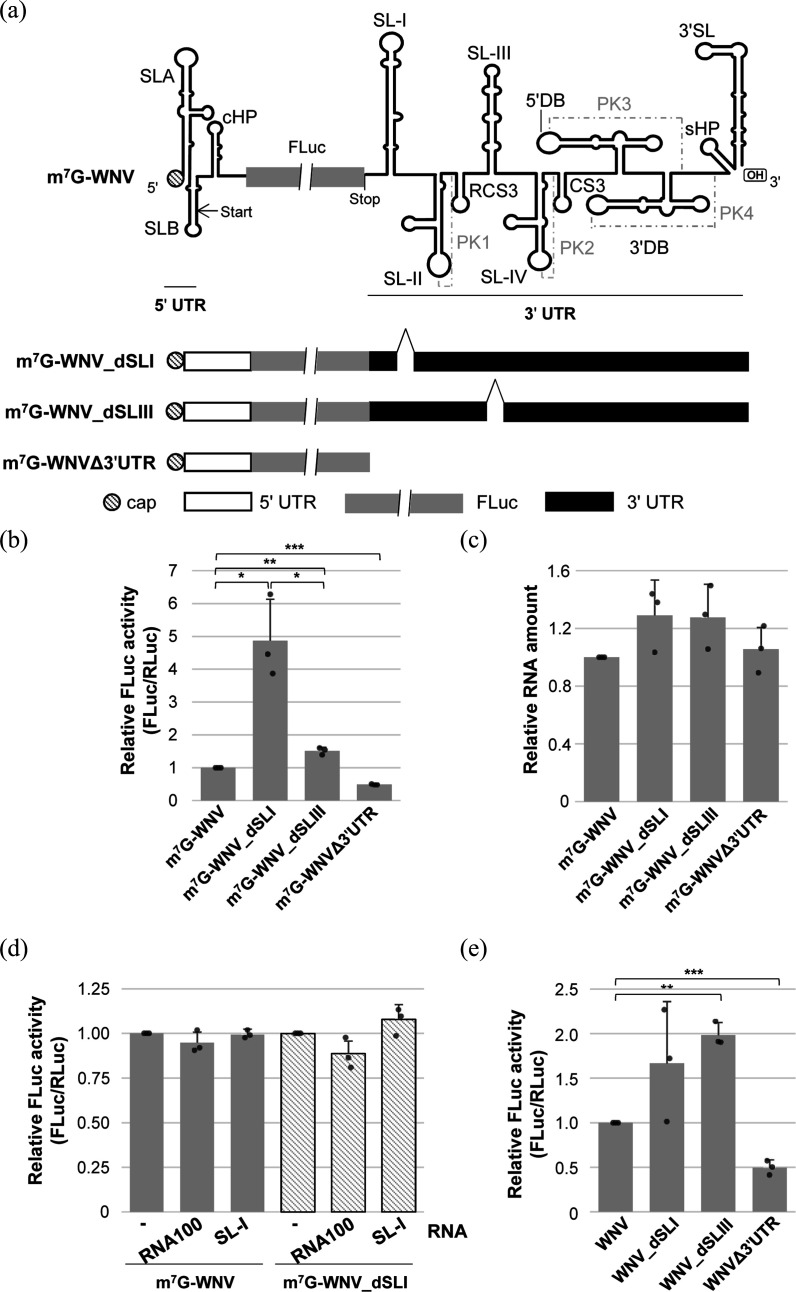
Inhibition of translation by the WNV SL-I element. (**a**) Diagram of the subgenomic WNV constructs used in this study, including the main structural elements. RNA molecules harbour the FLuc coding sequence flanked by the WNV 5′ and 3′ UTR. Start and stop translation codons are indicated by arrows. Deleterious constructs for SL-I, SL-III or the whole 3′ UTR are shown in the lower panel. (**b**) Translation efficiency of the different constructs shown in (a). Vero cells were independently cotransfected with each RNA construct plus the m^7^G-RLuc. Translation activity was determined at 6 h post-transfection as the ratio FLuc/RLuc and referred to that obtained for the m^7^G-WNV RNA. (**c**) Intracellular stability of the RNA constructs shown in (a). Vero cells were transfected with the RNA constructs and total RNA was isolated at 6 h post-transfection. RT-qPCR was performed with specific primers for the FLuc coding sequence and the GAPDH mRNA. The results were analysed with the Bio-Rad CFX Manager (Bio-Rad). (**d**) Effect of the SL-I element on viral translation in trans. Vero cells were cotransfected with the m^7^G-WNV or the m^7^G-WNV-dSLI and the m^7^G-RLuc RNA in the absence or presence of a molar excess of RNA SL-I or RNA100. Translation activity was determined as described in (b). Values refer to those obtained in the absence of RNA SL-I or RNA100. (**e**) Effect of the WNV SL-I element in translation of uncapped constructs. The RNA constructs shown in (a) were synthesized in the absence of cap analogue and used to transfect Vero cells along with the m^7^G-RLuc RNA. Translation activity was measured as indicated in (b). Values are the mean of three independent experiments±sd. Dots indicate individual data point. Statistically significant differences were calculated by applying a non-parametric Mann–Whitney test and are indicated by asterisks. *P<0.05, **P<0.01 and ***P<0.001.

We had previously observed that the SL-III element of the WNV 3′ UTR negatively regulates translation in WNV [6]. Given that SL-I and SL-III were identified as duplicated elements, we investigated whether SL-I might play a redundant role with respect to SL-III. To this end, translation assays were conducted in cell culture using two RNA constructs (Fig. 2a): m^7^G-WNV, which encodes the FLuc protein, flanked by the 5′ UTR of WNV plus the cHP element and the complete WNV 3′ UTR [6] and m^7^G-WNV_dSLI, which shares the same genetic organization but is deficient in the SL-I element of the 3′ UTR. The previously described constructs m^7^G-WNV_dSLIII, lacking the SL-III element, and m^7^G-WNVΔ3′UTR, which lacks the entire 3′ UTR, were also included in the study as internal controls [6] (Fig. 2a). These constructs were 5′-capped (see Methods) and used to co-transfect Vero cells along with the m^7^G-RLuc mRNA in order to normalize transfection efficiency [6]. The latter RNA encodes the RLuc protein, and its translation is independent on both the 5′ and the 3′ WNV UTRs. Both FLuc and RLuc activities were measured four h post-transfection.

In agreement with our previous findings [6], the deletion of the SL-III element induced a significant increase in FLuc activity (*P*<0.01), whereas the entire 3′ UTR was required for efficient translation (Fig. 2b). Interestingly, analysis of the m^7^G-WNV_dSLI transcript revealed that deletion of SL-I also enhanced translation, indicating that SL-I functions as a translational repressor (*P*<0.05), similar to the duplicated SL-III element. Notably, the magnitude of this effect was more than three times greater for the m^7^G-WNV_dSLI transcript compared to the SLIII-deficient construct (*P*<0.05), suggesting that SL-I is a potent negative regulator of translation. Slight variations in RNA stability were not statistically significant (Fig. 2c), supporting that the translational activity observed for the different constructs under study is a specific effect.

To find out whether SL-I can exert its function in trans, Vero cells were co-transfected with m^7^G-WNV and m^7^G-WNV_dSLI RNAs alongside a transcript consisting of SL-I (positions 10,400–10,500) element or an unrelated RNA of similar size (RNA100) [32]. The m^7^G-RLuc RNA was also included to normalize transfection efficiency and to detect potential non-specific effects of SL-I on general cap-dependent translation. The results demonstrated that the addition of SL-I in trans did not induce changes in FLuc activity or affect general cap-dependent translation (Fig. 2d), indicating that SLI’s role in WNV translation is strictly *cis*-dependent.

Next, we hypothesized that the translational repression effect exerted by SL-I could be recapitulated in a cap-independent translational context. To test this, the constructs described above were synthesized in the absence of cap analogue to generate the transcripts WNV, WNV_dSLI, WNV_dSLIII and WNVΔ3′UTR. Vero cells were transfected with each of these RNAs along with the m^7^G-RLuc RNA, as described (see Methods). Consistent with our previous findings, efficient translation required the complete 3′ UTR, while SL-III acted as a negative regulator of translation (Fig. 2e). The magnitude of the effect exerted by these transcripts was comparable to that observed in a cap-dependent translation context. Surprisingly, the effect of SL-I was markedly attenuated, with no significant differences in translation efficiency compared to the WNV construct (Fig. 2e). These results suggest the existence of distinct control mechanisms mediated by the SL-III and SL-I elements in cap-dependent and cap-independent translation in WNV.

### The WNV SL-I element reduces translation efficiency in DENV

**Fig. 3.**
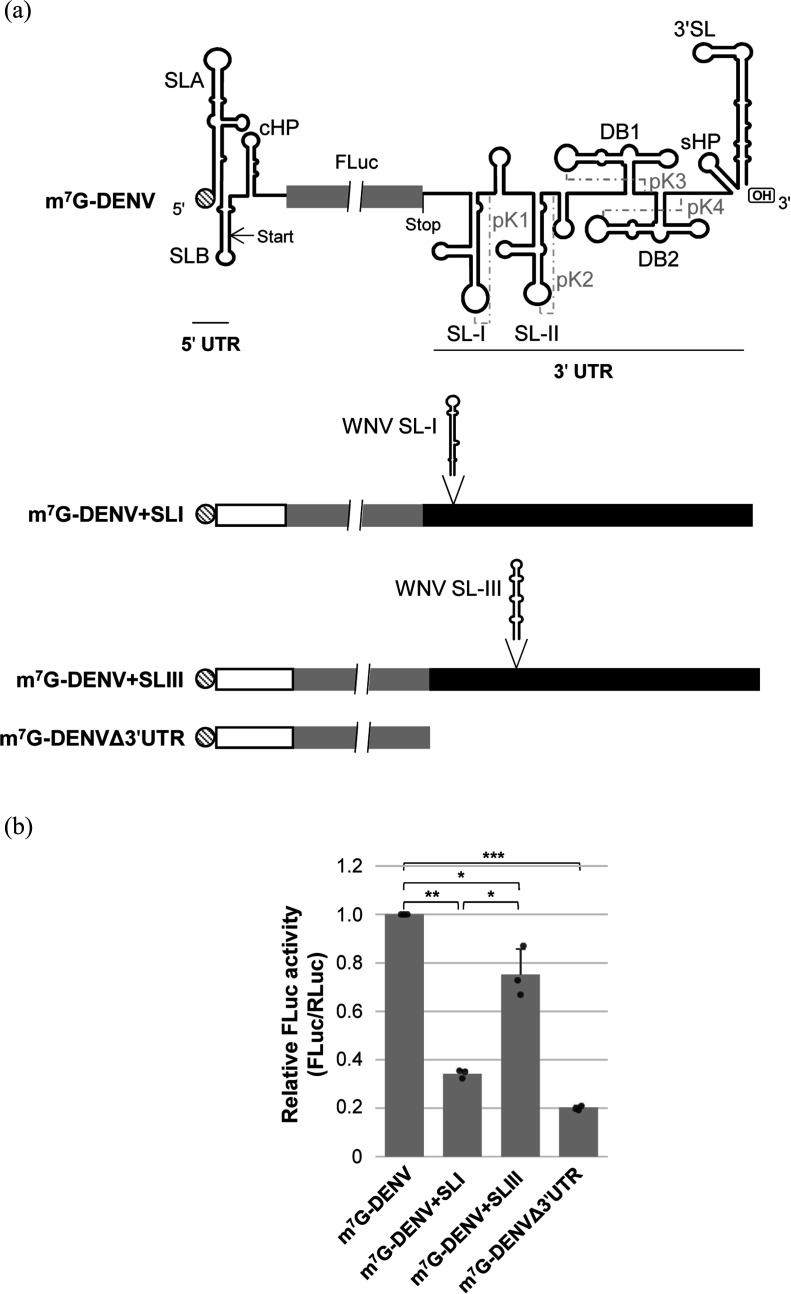
The WNV SL-I element negatively regulates DENV translation. (**a**) Diagram showing the RNA constructs used, harbouring the FLuc coding sequence flanked by the DENV 5′ and 3′ UTRs. The WNV SL-I and SL-III elements were included in homologous positions in the DENV 3′ UTR to those occupied in the WNV 3′ UTR to generate mutant constructs. (**b**) DENV translation efficiency in the presence of the WNV SL-I and SL-III elements. Vero cells were cotransfected independently with each of the above constructs plus the m^7^G-RLuc mRNA. Translation activity was detected as noted in Fig. 2(b). Data are the mean of three independent experiments±sd. Dots indicate individual data point. A Kruskal–Wallis test was performed to assess differences among the RNA constructs. Mann–Whitney U tests were subsequently applied as post hoc analyses for pairwise comparisons, with statistically significant differences indicated by asterisks: *P<0.05, **P<0.01 and ***P<0.001.

As mentioned above, domain I is the most variable tract of the 3′ UTR among orthoflaviviruses. Indeed, the SL-I and SL-III elements are absent in DENV, ZIKV and YFV, among others. To investigate whether the effects of WNV SL-I and SL-III could be extrapolated to heterologous molecular contexts, a set of subgenomic RNA constructs based on DENV was designed (Fig. 3a). The parental transcript, m^7^G-DENV, contains the 5′ and 3′ UTRs of DENV flanking the FLuc coding sequence. The translation start codon of the FLuc gene was removed to ensure translation initiation at the appropriate AUG codon in the 5′ UTR. From this construct, chimeric mutant RNAs were generated (Fig. 3a): m^7^G-DENV+SLI, which incorporates the WNV SL-I element at the 5′ end of the DENV 3′ UTR; m^7^G-DENV+SLIII, including the WNV SL-III element placed at the 3′ end of DENV PK1, a homologous position to that occupied by SL-III in the WNV 3′ UTR; and m^7^G-DENVΔ3′UTR, which lacks the 3′ UTR. All RNAs were synthesized in the presence of cap analogue and used to transfect Vero cells, as mentioned above, along with the transcript m^7^G-RLuc, in order to normalize transfection efficiency (see Methods). Consistent with previous observations [7], our results confirmed that the 3′ UTR of DENV is essential to achieve efficient viral translation (Fig. 3b). Importantly, both WNV SL-I and SL-III retained their translational repressor activity (Fig. 3b). Furthermore, as observed in the WNV model, SL-I exhibited a greater repressor activity than SL-III (*P*<0.05), reinforcing the idea that the molecular mechanisms underlying SL-I function differ from those of SL-III.

### SL-I influences multi-round translation without affecting the translation rate

The effect of SL-I as a translational repressor is likely due to a reduction in the number of translational events, i.e. a reduction in multi-round translation, resulting in decreased translation yield. This reduction may also be due to a lower translational rate, likely during the initiation step. To test this hypothesis, *in vitro* translation kinetic assays were performed using Vero cells S10 lysates. The RNA constructs m^7^G-WNV and m^7^G-WNV_dSLI were used as templates for protein synthesis, and the luciferase activity was measured *in situ* over the course of the translation reaction every 20 s. The collected data were used to determine the rate of the first translation round (*T*_1st_), as described [41]. To this end, the first derivative of luciferase activity values along time was fitted to a normal distribution, where the mean represents *T*_1st_ and the maximum value of the curve corresponds to protein synthesis at steady state.

**Fig. 4.**
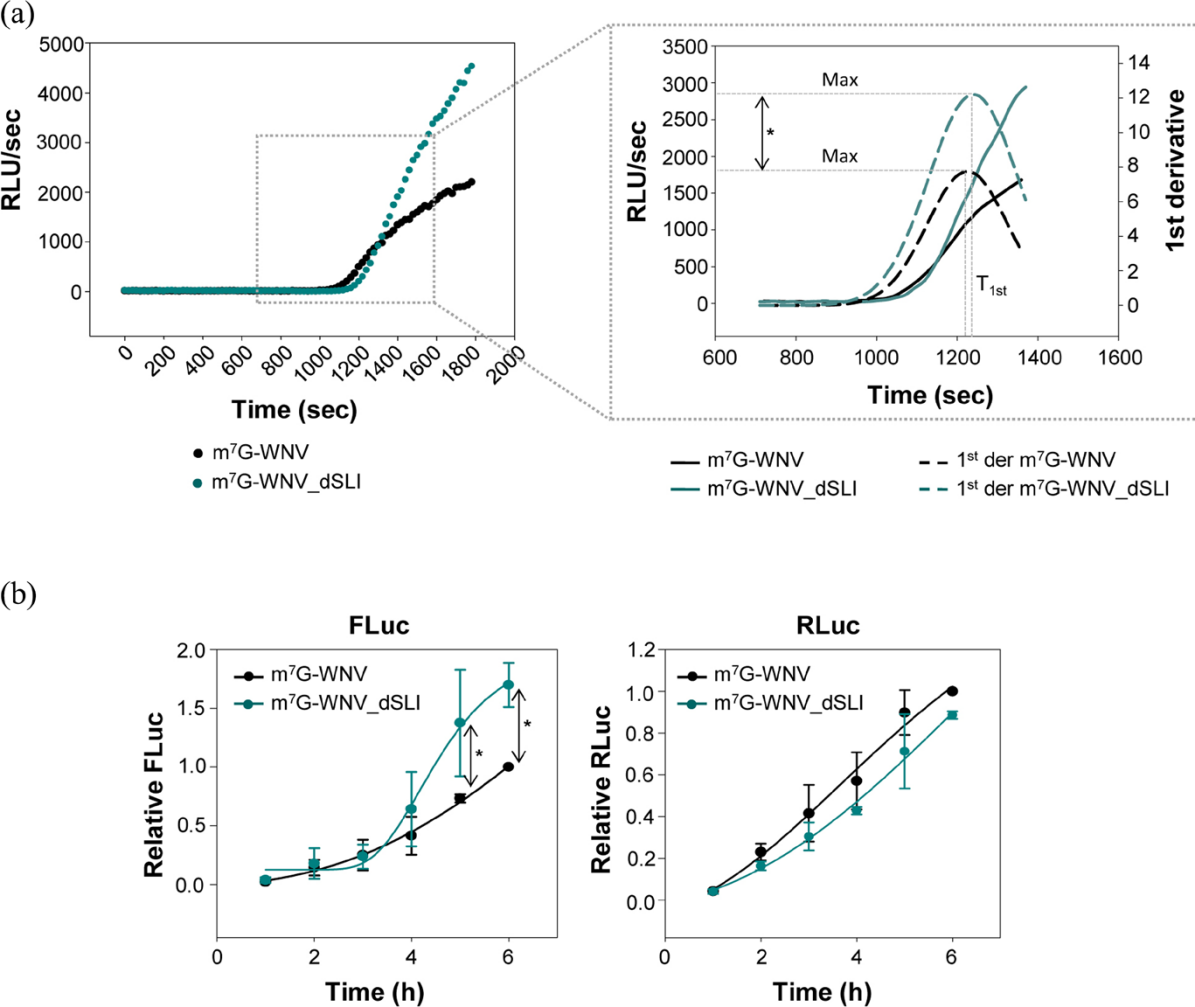
SL-I reduces the translation yield without modifying the translation rate. (**a**) Luciferase activity of the RNA constructs m^7^G-WNV and m^7^G-WNV_dSLI in S10 Vero cell lysates recorded at 20-s intervals. Data were fitted both to a Gompertz growing equation (left panel) as well as to a cumulative function of normal distribution (right panel), in order to calculate the yield of the reaction, the *T*_1st_ and the time at which translation reaches the maximum rate (*x*_0_). **(b**) Kinetic analysis of the translation activity for the m^7^G-WNV and m^7^G-WNV_dSLI RNA constructs in cell culture. Vero cells were cotransfected with m^7^G-WNV or m^7^G-WNV_dSLI plus the m^7^G-RLuc mRNA. Translation activity was determined at the indicated time points, and relative translation efficiency was calculated by referring the ratio FLuc/RLuc to that obtained for the control construct m^7^G-WNV at 6 h post-transfection. Data points were fitted to a Gompertz equation. Values are the mean of three independent experiments±sd. Statistically significant differences (*P*<0.05) were calculated by pairwise comparisons with a non-parametric Mann–Whitney U test and are indicated by an asterisk (*).

The results demonstrated that steady-state translation efficiency is significantly higher (*P*<0.05) in the absence of SL-I in S10 cell lysates (Fig. 4a, Table 1), consistent with prior observations in cellular models, thus confirming the robustness of the *in vitro* system. Similarly, protein synthesis efficiency during the first translation round was higher in the construct lacking SL-I (Fig. 4a, Table 1), but no significant variations in the *T*_1st_ parameter were observed in the absence of SL-I, suggesting that the rate of the process is not affected by the presence of SL-I at initial translation stages.

**Table 1.** Kinetic parameters obtained for the first round of *in vitro* translation

First round analysis		
**RNA**	Max*	***T*_1st_ (s**)
**m^7^G-WNV**	9.73±0.20	983.22±5.64
**m^7^G-WNV_dSLI**	17.08±2.22	1082.11±5.51

Max and *T*_1st_ parameters were obtained from the first derivative of luciferase activity across time, as described [41]. Data are the mean of three independent experiments±sd.

*Statistically significant variations (*P*<0.05).

Although the first-round translation rate remained unaffected, it seems likely that SL-I may regulate protein synthesis efficiency by controlling the rate of the process during subsequent rounds, thereby contributing to an overall global effect. To go further into this idea, the collected data were fitted to a Gompertz equation, *y* = *y*_0_ + a*e^(-(*x*_0_-*x*)/*b*)^, where *y* represents the translation efficiency, *y*_0_ is the baseline value, *a* is the amplitude or maximum growth value of the translation reaction, *x* is the time along the reaction, *x*_0_ is the time at which the reaction rate is maximal and *b* controls the rate at which the reaction reaches its maximum value. Surprisingly, the m^7^G-WNV_dSLI construct did not exhibit a significant increase in overall translation rate across the evaluated parameters (see Table 2), which discards that SL-I operates by reducing the global translation rate.

**Table 2.** Kinetic parameters for the *in vitro* translation assays in the steady state

Steady-state analysis			
**RNA**	a*	***x*_0_ (s**)	***b* (s**)
**m^7^G-WNV**	2633.23±132.67	1223.40±41.39	172.63±9.41
**m^7^G-WNV_dSLI**	5148.61±504.48	1264.87±29.60	173.05±17.60

Constants were obtained from the Gompertz equation *y* = *y*_0_ + a*e(-(-)/). Data are the mean of three independent experiments±sd.

*Statistically significant variations (*P*<0.05).

The observation that SL-I does not appear to affect the global translation rate was further corroborated in a cellular model. To this end, Vero cells were transfected with the aforementioned constructs, m^7^G-WNV and m^7^G-WNV_dSLI, alongside the m^7^G-RLuc mRNA. Luciferase activity values were collected at various time points post-transfection, and the obtained data were fitted to a Gompertz equation. The results confirmed that the presence or absence of SL-I did not significantly influence the translation rate (Table 3, Fig. 4b). Meanwhile, the absence of SL-I promoted a significant increase (*P*<0.05) in luciferase activity at 5 and 6 h post-transfection. We cannot exclude slight changes in the process rate during the early stages of translation as detected for the *in vitro* assays (Table 1).

**Table 3.** Kinetic parameters obtained for the translation assays in cell culture

	*a*	*x*_0_ (h)	*b* (h)
**m^7^G-WNV**	1.23±0.19	4.44±1.06	3.15±0.68
**m^7^G-WNV_dSLI**	1.83±0.31	4.17±0.20	0.94±0.33

Constants were obtained from the Gompertz equation *y* = *y*_0_ + a*e(-(-)/). Data are the mean of three independent experiments±sd.

Taken together, these results suggest that SL-I impairs the efficiency of multi-round translation without interfering with the kinetics of the process during early stages.

### SL-I influences the recruitment of the 40S subunit at the 5′DB element

Previous studies have demonstrated that the 5′DB element and the PK3–PK4 structural motif are essential components for the binding of the 40S ribosomal subunit and the translational control exerted by the WNV 3′ UTR [6,8]. To investigate whether the interaction between the 40S subunit and the 3′ UTR could be modulated by the SL-I element via 5′DB and PK3-PK4, the reactivity pattern of this interaction for NMIA reagent was analysed in the presence and absence of SL-I. For these experiments, the transcripts m^7^G-WNV and m^7^G_WNV_dSLI RNA described above were probed by SHAPE in the presence of the 40S subunit (see Methods). In parallel, these assays were performed in the absence of the 40S subunit in order to evaluate potential reactivity disruptions derived from the absence of SL-I. The SHAPE analysis targeted the terminal 200 nts of the 3′ UTR, including the 5′DB element and the PK3-PK4 structure [8]. The reactivity values were subsequently normalized according to the previously described procedure [39], which enables comparison across different molecules while mitigating any bias arising from variations in the overall molecular reactivity or subsequent processing.

**Fig. 5.**
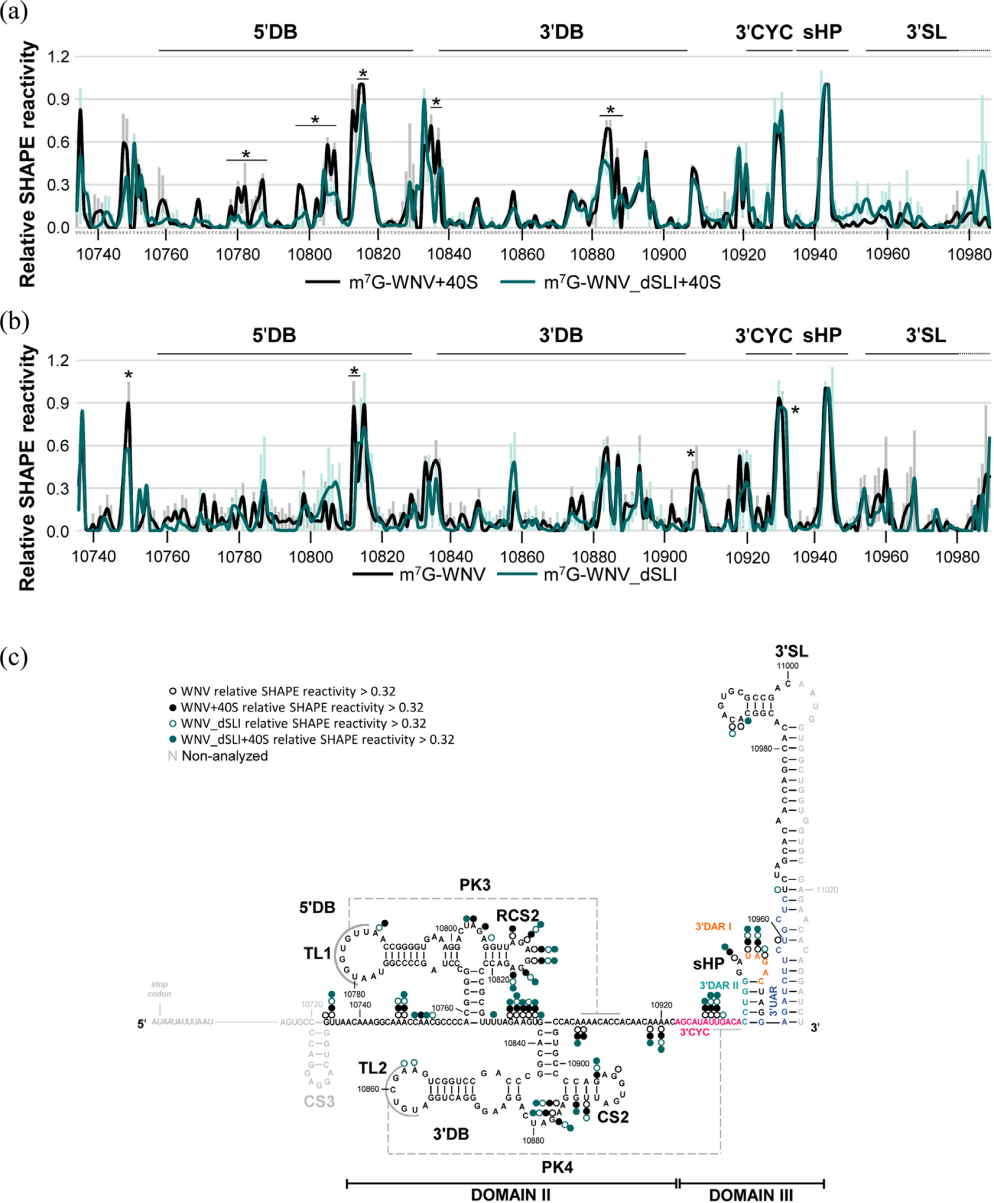
SL-I influences the positioning of the 40S ribosomal subunit in the WNV 3′ UTR. (**a**) The binding of the 40S ribosomal subunit was analysed in the WNV 3′ UTR by SHAPE, with (black line) or without SL-I (cyan line). Relative NMIA reactivity was calculated and further normalized as described [33,39]. Structural elements and sequence motifs are indicated. (**b**) SHAPE analysis of the nucleotides 10,740–10,980 of the WNV 3′ UTR in the transcripts m^7^G-WNV (black line) and m^7^G-WNV_dSLI (cyan line). Relative reactivity values were calculated as described in (a). (**c**) Summary of the SHAPE analyses shown in (a) and (b). Nucleotides exhibiting a relative reactivity >0.4 units are indicated by open circles (−40S) or filled circles (+40S). Numbering refers to positions indicated in Fig. 1. Values are the mean of three independent experiments±sd. *Significant differences in relative NMIA reactivity (*P*<0.05) calculated by non-parametric Mann–Whitney and Kruskal–Wallis tests.

The relative reactivity profiles for both RNAs in the presence of the 40S ribosomal subunit were largely overlapping across the analysed region, with the exception of nucleotides located in 5′DB and 3′DB (U_10780_–G_10781_, A_10797_–A_10798_, U_10804_–A_10807_, A_10813_–A_10815_, A_10835_–U_10837_ and A_10883_–A_10884_; Fig. 5a, c). At these positions, the absence of SL-I led to a significant decrease in reactivity, which could be due to an enhanced binding efficiency of the 40S subunit. The relative reactivity profiles for both transcripts in the absence of the 40S subunit were largely identical (Fig. 5b), with the exception of single positions A_10754_, A_10817_–G_10818_, G_10821_, A_10912_ and G_10938_ (Fig. 5b, c). These structural data reinforce our findings and point to SL-I as a relevant partner in the regulation of the 40S recruitment by the WNV 3′ UTR and its role in viral translation.

## Discussion

The translation regulation in WNV results from the combined action of different RNA elements located in the genomic UTRs. Specifically, from those of the 3′ end, elements within domains II and III function as translation enhancers, whereas the SL-II, SL-III and SL-IV elements, located in domain I, appear to act as negative regulators of protein synthesis [6]. The present manuscript demonstrates that the SL-I element within domain I also operates as a cis-acting translational repressor in the steady state of viral translation, placing domain I of the WNV 3′ UTR as a negative partner in the regulation of viral protein synthesis.

Domain I within the orthoflaviviruses 3′ UTR shows great heterogeneity in size and sequence [42,45]. Such heterogeneity could be related to the duplication of elements SL-I–SL-II–PK1–RCS3 and SL-III–SL-IV–PK2–CS3 [46]. Therefore, it seems reasonable to assume that redundant functions for the two duplicated units can diverge to amplify the functional versatility of the 3′ UTR. Our data show that SL-I is a more efficient translational repressor than SL-III, not only in WNV but also in the heterologous molecular context of the DENV 3′ UTR (Figs2  3). Nonetheless, given that SL-I and SL-III are not conserved elements across orthoflaviviruses, it is unlikely that their roles in translation regulation are essential for viral viability, although our data suggest that they appear to contribute to the regulation of the WNV cycle. Finally, it must be noted that the differences in translational activity exhibited by SL-I and SL-III cannot be attributed to variations in the stability of the tested constructs (Fig. 2). This finding aligns with previous studies on other members of the family *Flaviviridae*, which have demonstrated that the deletion of the 3′ UTR or its constituent elements does not result in significant changes in RNA stability that would impact translational activity [26,47,49].

The underlying mechanism of translational regulation seems to differ between SL-I and SL-III: while the negative regulation exerted by SL-III is preserved in cap-dependent and cap-independent translation, SL-I effect is not detected in uncapped transcripts (Fig. 2e). This observation would support the notion that the functional roles of these two elements have diverged throughout the evolution of the clade, as has been proposed for other structural elements [4]. Notably, the WNV_dSLI RNA displays high variability, which seems to be an intrinsic property of this molecule, likely reflecting heterogeneity in its activity. Such variability may arise from multiple cellular factors [50], including differences in metabolic state, limiting levels of essential cofactors required for RNA function, cell-cycle heterogeneity and even nonspecific activation of innate immune sensors, such as PKR, RIG-I or MDA5, which are involved in the regulation of translation.

The functional divergence between SL-I and SL-III could be influenced by distinct three-dimensional architectures. SL-III exhibits autonomous folding, forming a structurally compact element, whereas SL-I is proposed to serve as a flexible spacer that facilitates the proper folding of the remaining elements within the 3′ UTR [16,51]. The high structural flexibility of SL-I enables conformational rearrangements, which could allow it to function as a conditional regulator responsive to environmental cues or viral life cycle stages. In fact, beyond its role as a negative regulator of translation, SL-I induces the accumulation of viral genomes [52].

During orthoflavivirus infection, stress granules assemble and sequester translation-related factors as well as 43S and 48S ribosomal complexes [53]. Given that the WNV 3′ UTR recruits the 40S ribosomal subunit at SL-III and 5′DB to control viral translation, it is likely that specific regulatory elements within the viral genome modulate this interaction to regulate protein synthesis under adverse conditions. Indeed, preliminary data from our group suggest that deletion of the SL-I element may increase both the affinity and the rate of 40S subunit recruitment at the lower-affinity binding site (see Fig. S1 and Table S2). Further studies are required to determine whether these observations support a molecular mechanism in which SL-I regulates translation by modulating the rate and efficiency of 40S subunit binding to the 3′ UTR. This hypothesis agrees with the kinetic analysis of the translation process, showing that SL-I does not influence the rate of protein synthesis, but it affects the efficiency of the process. This effect may be due to the reduced affinity of the 3′ UTR for the translational machinery in the presence of SL-I, with maximal impact observed during steady-state conditions. It must also be noted that protein factors related to translation regulation, distinct from those that form the ribosome, can also be involved in the activity exerted by SL-I [54].

The ability of SL-I to regulate translation at specific stages of the process may be linked to the establishment of intra- and intermolecular interactions with the 3′CYC sequence, thereby modulating the conformational equilibrium with PK4, which is critical for 40S ribosomal subunit recruitment [33]. SHAPE analysis revealed a modest but statistically significant reduction in the reactivity profile at position G_10931_ within the 3′CYC sequence motif, and also at positions A_10750_, A_10813_–G_10814_ and A_10908_ (Fig. 5b, c). This profile likely reflects subtle structural rearrangements in the 3′ UTR mediated by SL-I within the affected elements, which could be involved in the switch between different local dynamics, as mentioned above. However, these changes appear insufficient to fully account for the observed effects on translation and 40S subunit recruitment. The possibility of additional SL-I-induced conformational changes in other 3′ UTR elements, beyond those investigated here, cannot be ruled out. Nevertheless, significant structural rearrangements would be expected to promote pronounced changes in the overall SHAPE reactivity pattern, or at least in large nucleotide stretches, which were not observed. Hence, the activity exerted by SL-I seems to be independent of dramatic conformational changes within the 3′ UTR.

Based on the presented data and previously published findings [8], we propose that during the initial stages of viral translation, the 3′ UTR facilitates the recruitment of the 40S ribosomal subunit and promotes its positioning at the 5′ UTR, thus increasing the initial translation efficiency. In the steady state, the accumulation of translational machinery at the 3′ end of the ORF may impede the delivery of the 40S subunit to the 5′ UTR through a mechanism involving the SL-I element. This mechanism is likely associated with conformational changes in the 5′DB, PK3 and PK4 structures. The reduction in translational efficiency during the later stages may facilitate the release of critical RNA elements required for the initiation of replication, including SLA at the 5′ end and 5′DB, sHP and 3′SL within the 3′ UTR.

In summary, these results provide, for the first time, evidence of the role of SL-I in translational control, demonstrating that it acts as a negative regulator of protein synthesis in WNV. This effect may be associated with SL-I mediated modulation of the interaction between the 3′ UTR and the 40S ribosomal subunit, resulting in reduced affinity and slower binding kinetics. Furthermore, the observation that SL-I is functional only in cis supports the notion that the 3′ UTR fulfils a multifunctional role, wherein its distinct structural elements operate in a coordinated manner to regulate the transitions between different stages of the viral cycle.

## Supplementary material

10.1099/jgv.0.002268 Uncited Table S1.

## Abbreviations

DB: dumbbell
DENV: dengue virus
MEM: Minimum Essential Medium
NMIA: *N*-methylisatoic anhydride
RT-qPCR: reverse transcription-quantitative PCR
SHAPE: selective 2′-hydroxyl acylation analysed by primer extension
sHP: small hairpin
3′SL: 3′ stem-loop
WNV: West Nile virus
YFV: Yellow Fever virus
ZIKV: Zika virus
