# Supplementary material for: The SL-I structural element of the 3′ UTR region of West Nile virus participates in the regulation of viral translation
Source: J Gen Virol. 2026 May 28;107(5):002268. doi: 10.1099/jgv.0.002268 (PMC13224872; doi:10.1099/jgv.0.002268)
Supplement: Uncited Table S1. [file jgv-107-02268-s001.pdf]

SUPPLEMENTARY MATERIAL

The SL-I structural element of the 3' UTR region of WNV participates in the regulation of viral translation.

Cristina Romero-López*, Pilar Bueno-Arribas and Alfredo Berzal-Herranz*

Instituto de Parasitología y Biomedicina "López-Neyra", IPBLN-CSIC. PTS Granada, Av. del Conocimiento 17, 18016, Armilla (Granada). Spain

Supplementary Table 1. Primer sequences used in this study

Procedure	Primer	Sequence (5’-3’)
Site-directed mutagenesis to generate pGLWNV_dSLI	WNV-10496	TGAGGAGAAAGTCAGGCCGGGA
	asFLuc	ATTACACGGCGATCTTTCCGC
qPCR	qFLuc-F	ACTGGGACGAAGACGAACAC
	qFLuc-R	GGCGACGTAATCCACGATCT
SHAPE assays	as3’WNV	AGATCCTGTGTTCTCGCACCA

Supplementary Figure 1

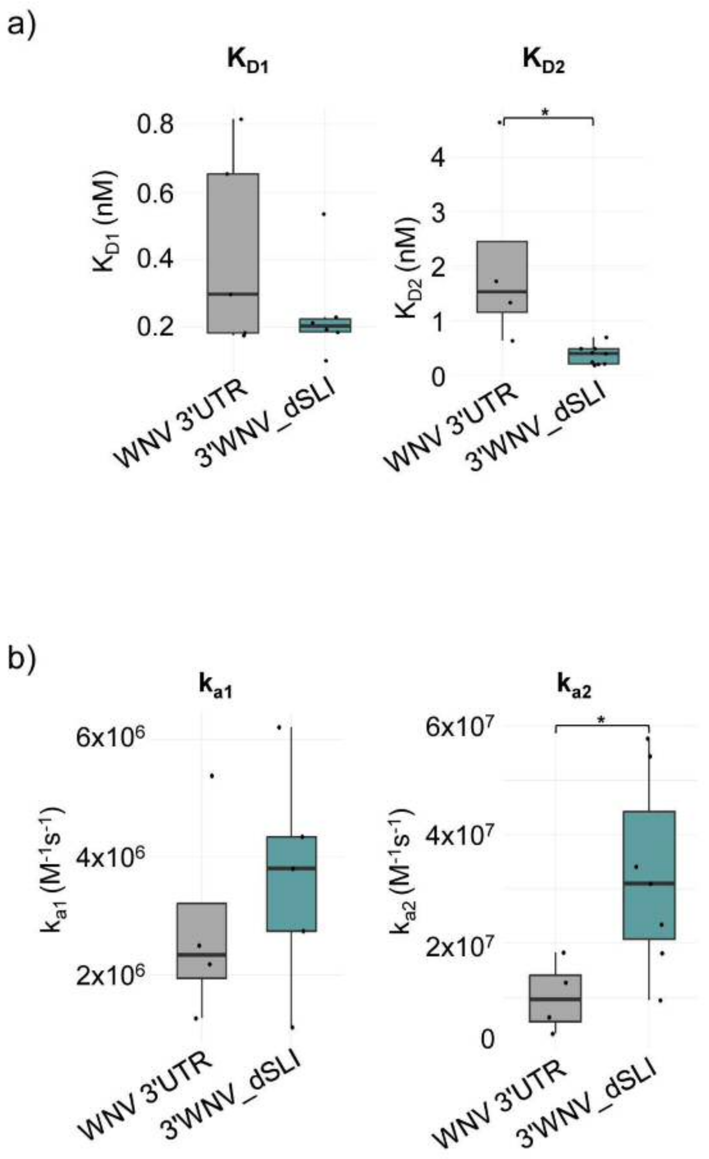

Supplementary Figure 1. SL-I affects the binding kinetics of the 40S ribosomal subunit to the WNV 3' UTR. a) and b) Box plots showing the K_D and k_a values for the interaction WNV 3' UTR-40S at two binding sites, 1 and 2. The RNA constructs WNV 3' UTR or WNV_dSLI were incubated in the presence of a molar excess of the 40S ribosomal subunit. Binding efficiency was detected by biolayer interferometry using Octet®RED-96 equipment (Sartorius AG). Assays were performed as described in supplementary methods. Constants were calculated by using the Octet Data Analysis software (Sartorius AG). Data were visualized as box plots and significant differences ($p < 0.05$) were calculated by

the non-parametric Mann-Whitney U test. (*), statistically significant differences. Sample size: at least 4 and 5 independent experiments were used to calculate the kinetic parameters of the WNV 3'UTR RNA and the 3'WNV_dSLI RNA, respectively.

The affinity constants yielded K_D values in the low nanomolar range for both theoretical binding sites in the WNV 3' UTR RNA, with a ten-fold variation between sites 1 and 2 (Table S2). This result corroborates previous observations suggesting the presence of two distinct sites with different affinities [8]. Notably, the deletion of the SL-I element resulted in a significant increase in affinity for the 40S subunit at the lower-affinity binding site (site 2), yielding near 10-fold reduction in K_D (K_{D2}; Figure S1 and Table S2), whereas the K_D value for the higher-affinity site remained unchanged (K_{D1}). This behavior was consistent for the association rate constant, k_a: while no significant changes were observed in k_a for site 1 (k_{a1}), the k_a value for site 2 (k_{a2}) increased by 3-fold, indicating a higher binding rate of the 40S subunit at this interaction site. No changes were detected in the dissociation rate constant, k_{dis}, for either of the two theoretical sites, suggesting that the effect of SL-I on 40S binding is linked with the association step.

Supplementary Table 2. Affinity and rate constants for the interaction between the 3' UTR and the 40S ribosomal subunit in the absence and presence of the SL-I element.

	WNV 3' UTR	WNV_dSLI
**K_{D1} (nM)**	0.42 ± 0.29	0.24 ± 0.15
**K_{D2} (nM)**	2.09 ± 1.77	0.36 ± 0.17 (*)
**k_{a1} ($M^{-1}s^{-1}$)**	$2.83x10^6$ ± $1.78x10^6$	$3.64x10^6$ ± $1.89x10^6$
**k_{a2} ($M^{-1}s^{-1}$)**	$1.02x10^7$ ± $6.69x10^6$	$3.26x10^7$ ± $1.79x10^7$ (*)
**k_{dis1} (s^{-1})**	$5.72x10^{-4}$ ± $3.30x10^{-4}$	$5.98x10^{-4}$ ± $3.33x10^{-4}$
**k_{dis2} (s^{-1})**	$1.44x10^{-2}$ ± $6.25x10^{-3}$	$1.14x10^{-2}$ ± $1.32x10^{-2}$

(*) indicates significant differences (p<0.05). 1 and 2 correspond to the data obtained for sites 1 and 2 respectively. Data are the mean of three independent experiments ± standard deviation. A non-parametric Mann-Whitney U test was applied to identify statistically significant variations (p < 0.05), indicated by (*).

Supplementary Methods

Binding assays using biolayer interferometry (BLI)

Biolayer interferomentry technique was applied to analyze the 40S recruitment by the 3' UTR using Octet®RED-96 equipment (Sartorius, Germany). Opaque-bottom 96-well plates were used as the support for solutions and high-precision SAX2 streptavidin biosensors (Sartorius AG), which were hydrated for 10 minutes prior to use to remove their protective sucrose coating. The general assay protocol was established using the Octet Data Acquisition software: (1) Baseline: initial 1-minute baseline to equilibrate the system in binding buffer (20 mM HCl; 60 mM NH_4Cl; 7.5 mM MgOAc, 0.5 mM EDTA, 6 mM β-Mercaptoethanol, 0.1% (w/v) BSA, 0.02% (v/v) Tween-20 pH 7.5) to reduce non-specific interactions of the RNAs and/or the 40S ribosomal subunit with the biosensor surface. (2) Loading: biotinylated RNA transcripts, b-WNV 3'UTR and b-3’WNV_dSLI, were independently immobilized onto the biosensors during 10 min. The optimal immobilization concentration for each RNA was previously determined through titration assays. (3) Wash: a 1-min wash step in binding buffer was performed to remove unbound transcripts. (4) Baseline 2: second baseline acquisition for 5 minutes in binding buffer. (5) Association: immobilized RNAs were exposed to a range of increasing concentrations of the 40S ribosomal subunit (0 nM-25 nM) during 10 min in binding buffer. (6) Dissociation: a 10-minute dissociation phase by incubation in binding buffer. Each assay was performed in triplicate at 37°C with shaking 1000 rpm. The obtained data were processed using the Octet Data Analysis software. During the association and dissociation phases, data correction was applied, and the resulting sensorgrams were fitted to a 2:1 heterogeneous ligand binding model. This analysis enabled the determination of the kinetic and affinity constants for 40S subunit recruitment mediated by the different RNAs.

We are deeply grateful to Dr. Mario Delgado for providing us with the SAX2 streptavidin biosensors.
